# A multicenter, longitudinal survey of headaches and concussions among youth athletes in the United States from 2009 to 2019

**DOI:** 10.1186/s10194-022-01528-3

**Published:** 2023-02-08

**Authors:** Muhammad Ali, Nek Asghar, Theodore Hannah, Alexander J Schupper, Adam Li, Nickolas Dreher, Muhammad Murtaza-Ali, Vikram Vasan, Zaid Nakadar, Husni Alasadi, Anthony Lin, Eugene Hrabarchuk, Addison Quinones, Lily McCarthy, Zerubabbel Asfaw, Jonathan Dullea, Alex Gometz, Mark Lovell, Tanvir Choudhri

**Affiliations:** 1 Department of Neurosurgery, Icahn School of Medicine at Mount Sinai, 10021 NY, USA; 2 Department of Neurosurgery, Lewis Katz School of Medicine at Temple University, 19140 Philadelphia, PA USA; 3 Department of Neurosurgery, University of Rochester School of Medicine and Dentistry, 14642 Rochester, NY USA; 4 Department of Medicine, Columbia University Vagelos College of Physicians and Surgeons, 10032 NY, USA; 5 Department of Anthropology, State University of New York at Binghamton, 13902 NY, USA; 6 Department of Neurosurgery, State University of New York Downstate Health Sciences University, 11203 NY, USA; 7 Department of Pathology, Joan & Sanford I. Weill Medical College of Cornell University, 10021 NY, USA; 8 Concussion Management of New York, 10021 NY, USA; 9 Department of Neurology, The University of Pittsburgh Medical Center, 15260 Pittsburgh, PA USA

**Keywords:** chronic headache, concussion, youth athletes

## Abstract

**Objective/ background:**

Chronic headaches and sports-related concussions are among the most common neurological morbidities in adolescents and young adults. Given that the two can overlap in presentation, studying the effects of one on another has proven difficult. In this longitudinal study, we sought to assess the relationship between chronic headaches and concussions, analyzing the role of historic concussions on chronic headaches, as well as that of premorbid headaches on future concussion incidence, severity, and recovery.

**Methods:**

This multi-center, longitudinal cohort study followed 7,453 youth athletes who were administered demographic and clinical surveys as well as a total of 25,815 Immediate Post-concussion Assessment and Cognitive Testing (ImPACT) assessments between 2009 and 2019. ImPACT was administered at baseline. Throughout the season concussions were examined by physicians and athletic trainers, followed by re-administration of ImPACT post-injury (PI), and at follow-up (FU), a median of 7 days post-concussion. Concussion incidence was calculated as the total number of concussions per patient years. Concussion severity and recovery were calculated as standardized deviations from baseline to PI and then FU in Symptom Score and the four neurocognitive composite ImPACT scores: Verbal Memory, Visual Memory, Processing Speed, and Reaction Time. Data were collected prospectively in a well-organized electronic format supervised by a national research-oriented organization with rigorous quality assurance. Analysis was preformed retrospectively.

**Results:**

Of the eligible athletes, 1,147 reported chronic headaches (CH) at the start of the season and 6,306 reported no such history (NH). Median age of the cohort was 15.4 ± 1.6 years, and students were followed for an average of 1.3 ± 0.6 years. A history of concussions (OR 2.31, *P* < 0.0001) was associated with CH. Specifically, a greater number of past concussions (*r*^2^ = 0.95) as well as concussions characterized by a loss of consciousness (*P* < 0.0001) were associated with more severe headache burden. The CH cohort had a greater future incidence of concussion than the NH cohort (55.6 vs. 43.0 per 100 patient-years, *P* < 0.0001). However, multivariate analysis controlling for demographic, clinical, academic, and sports-related variables yielded no such effect (OR 0.99, *P* = 0.85). On multivariable analysis the CH cohort did have greater deviations from baseline to PI and FU in Symptom Score (PI OR per point 1.05, *P* = 0.01, FU OR per point 1.11, *P* = 0.04) and Processing Speed (OR per point 1.08, *P* = 0.04), suggesting greater concussion severity and impaired symptomatic recovery as compared to the NH cohort.

**Conclusion:**

A history of concussions was a significant contributor to headache burden among American adolescents and young adults. However, those with chronic headaches were not more likely to be diagnosed with a concussion, despite presenting with more severe concussions that had protracted recovery. Our findings not only suggest the need for conservative management among youth athletes with chronic headaches, they also indicate a potential health care gap in this population, in that those with chronic headaches may be referred for concussion diagnosis and management at lower rates than those with no such comorbidity.

**Supplementary Information:**

The online version contains supplementary material available at 10.1186/s10194-022-01528-3.

## Introduction

Chronic headaches are among the most common neurologic morbidities in adolescents. Chronic headache refers to headaches occurring every other day for at least three months [1, 2]. It is a persistent and debilitating condition, effecting physical health, mental well-being, academic performance, and student lifestyle. Recent estimates suggest adolescent headache prevalence of up to 80% [3, 4] and chronic headache prevalence of 3–6% [1, 2]. Multiple risk factors for chronic headaches among adolescents and young adults have been reported, including female gender, obesity, psychiatric comorbidities, alcohol and caffeine use, low socioeconomic status, and family history [5, 6]. Fully characterizing modifiable risk factors for chronic headaches and minimizing their effects is an important public health goal. Though chronic headaches are recognized as a common sequela of concussions [7], a history of concussion has not been included in large-scale surveys exploring risk factors of adolescent headaches [5, 6].

Sports-related concussion is the leading cause of adolescent injury in the United States, with an estimated 1.6 to 3.8 million annual injuries, making it a major public health concern [8]. Though preliminary studies have reported correlations between headaches and concussions [9–11], understanding the exact nature of the relationship has proven difficult given the similarities in presentation [12, 13]. It is possible that concussions contribute significantly to the national adolescent headache burden [12]. It is also possible that chronic headaches are a risk factor for future concussions and impaired recovery thereafter [13]. Therefore, we sought to longitudinally examine the relationship between chronic headaches and sports-related concussion. First, we assessed the role of historic concussions on chronic headaches reported at the beginning of the season. Then we sought to quantify the extent to which chronic headaches contributed to the future risk for concussion and protracted recovery during the course of the season. In doing so, we hoped to clarify not only the directionality of the relationship between the two conditions but also the current state of their clinical management.

Our multi-center, longitudinal study followed youth athletes in several states for an average of 1.3 years from 2009 to 2019. Our cohort includes patients with multiple sporting backgrounds and ranging in age from young adolescence (12, 13) to late adolescence (14–17) and into young adulthood (8–22). Chronic headaches were diagnosed at the start of the season. We used demographic and clinical surveys at baseline to quantify salient risk factors for chronic headaches. During the season, on field head trauma assessments were used for concussion incidence calculations. We also employed Immediate Post-concussion Assessment and Cognitive Testing (ImPACT) administered at baseline, post-injury, and follow-up a median of 7 days post-concussion to track headache and concussion severity through the injury and recovery process. In doing so, we hoped to better characterize the relationship between persistent headaches and concussions among youth athletes, at a time when both are more prevalent than before. We hypothesized that chronic headaches would be associated with both a history of concussion as well as with an increased risk for future concussions, greater concussion severity, and impaired concussion recovery.

## Methods

### Design, participants, and procedures

The study includes 7,453 student-athletes who reported to multiple concussion centers in four American cities from July 2009 to June 2019. Participants were selected from 9,226 total students [Supplementary Fig. 1]. Inclusion criteria included age ≥ 12 and ≤ 22, academic enrollment at a high school or college program affiliated with a participating concussion center, and at least one season of athletic participation in a school-affiliated sport including football, soccer, lacrosse, gymnastics, cheerleading, wrestling, martial arts, water polo, or diving. Exclusion criteria included participation in a non-school-affiliated sport, such as archery or skiing, incomplete baseline demographic data or invalid ImPACT results at baseline, post-injury, or follow-up.

**Fig. 1:**
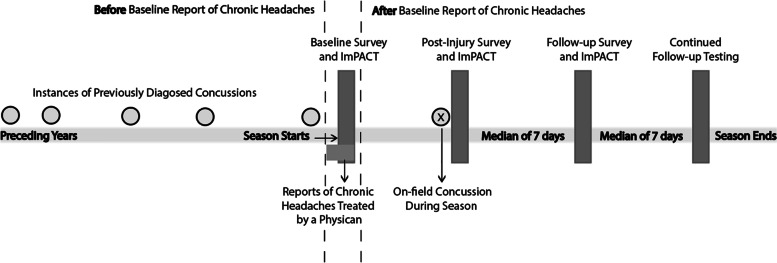
Study Design Organized and Supervised by ImPACT Applications Inc. Student-athletes with significant medical and sport-related history complete a baseline survey and ImPACT testing at the beginning of the season. Students are readministered ImPACT post-injury and at subsequent follow-up visits a median of 7 days apart

Data were collected at baseline at the beginning of the season, and included demographic information, medical history, a Post-Concussion Symptom Scale (PCSS) survey [14], as well as 39 separate neurocognitive tests on ImPACT [15]. Trained clinicians administered the assessments as part of a comprehensive clinical examination and interview. Throughout the season students received standardized care, including on-field head injury assessment by physicians, physiotherapists, and athletic trainers. Concussions were defined as blunt trauma to the head or face causing a rapid alteration of mental status and/ or the appearance of multiple symptoms not present before the injury, including headaches, dizziness, nausea, vomiting, and blurred vision [16]. Patients were followed longitudinally at concussion centers, with re-administration of the PCSS survey and ImPACT post-injury (PI) and at follow-up (FU), a median of 7 days after concussion to track the severity and the recovery of symptoms and neurocognitive dysfunction over time. After the initial follow-up visit patients presented to subsequent follow-up appointments, approximately 5 to 7 days apart, until they reached preinjury status or were deemed symptom free. Data were primarily employed for clinical care. Concussion centers also had agreements with ImPACT Applications to prospectively collect data through an electronic data collection system that was established and directly supervised by the organization so that data could be purposed for research.The participants’ legal guardians were aware that the data may be used in future research studies and provided informed parental consent with child assent. The data were deidentified before acquisition, and retrospective analysis of the data set was approved by the Institutional Review Board. The study adheres to the STROBE reporting guidelines [17] for observational studies [Supplementary Material]. A schematic of the study design is provided in Fig. 1.

### Measures

#### Demographic information and medical history

Participants provided demographic data including age, grade level, gender, sport played, primary on-field position, years of experience in the sport, number of games missed due to a history of concussion, history of special education and/ or speech therapy, and primary spoken language. Medical history data included previous “number of times diagnosed with a concussion,” characteristics of historic concussions, including confusion, anterograde amnesia, retrograde amnesia, and loss of consciousness, neuropsychiatric comorbidities, including chronic headaches, depression/ anxiety, attention deficit hyperactivity disorder, diagnosed learning disorder, dyslexia, autism, epilepsy, and substance use disorder, headache classification, including post-traumatic, migraine, and of unspecified classification, and generic names of prescribed medications. Students were specifically asked whether they were receiving “treatment for headaches by a physician.”

#### Concussion symptoms

The PCSS survey was employed to quantify symptom burden throughout the pre and postinjury periods. The PCSS survey includes 22 symptoms categorized into migraine, cognitive, neuropsychiatric, and sleep clusters. Each symptom is rated on a 7-point Likert scale from 0 (nonexistent) to 6 (severe), for an overall composite Symptom score ranging from 0 to 132. The survey has previously been validated in adolescent athlete populations [14].

#### Neurocognitive performance

ImPACT was employed to assess neurocognitive performance in verbal recognition memory, visual working memory, visual processing speed, reaction time, attentional processes, numerical sequencing, and learning. The ImPACT neurocognitive test requires completion of 6 cognitive subtest modules that traditionally generate four composite scores, including Verbal Memory, Visual Memory, Processing Speed, and Reaction Time. ImPACT is an extensive test, usually taking 25–30 min to complete. The assessment has previously been validated in adolescent athlete populations [15].

#### Concussion incidence

Incidence of concussion was calculated as the total number of concussions per total number of person-years. Person-years were determined per Centers of Disease Control and Prevention (CDC) guidelines [18]. In short, patients were required to have a baseline test to be considered at risk for concussion, with person years being calculated as the time difference between a patient’s baseline test and either their PI test or a subsequent baseline test if no injury was reported for that academic year. ImPACT baseline tests are reported to be stable for 2 years within the adolescent population, capping each baseline test at 2 person-years [19, 20]. Those with no further ImPACT testing after an initial baseline test were considered lost to follow-up and contributed 1 person-year.

#### Concussion severity and recovery

As previously described [21–24], deviations from baseline to PI and then FU in composite Symptom score and the four ImPACT composite scores were used to track the severity and recovery of symptoms and neurocognitive dysfunction post-concussion. Deviations were standardized as the deviation between two subsequent baseline tests of healthy control participants at the 80% confidence interval (S_diff_) [25]. For the Symptom Score composite the historic S_diff_ is 9.18, for Verbal Memory 8.75, for Visual Memory 13.55, for Processing Speed 4.98, and for Reaction Time 0.06.

Though clinical assessment is the gold-standard for severity and recovery, the CDC recommends physicians to use validated symptom scales like the PCSS (Level B recommendation) and age-appropriate cognitive testing (level C recommendation) like ImPACT to assess recovery in children with concussion [16]. The CDC states that “Symptom scales and cognitive testing (including measures of reaction time) have the strongest evidence in terms of their contribution to predicting outcomes and assessing recovery.” [16].

### Data Analysis and Statistics

Participants were dichotomized into those with chronic headaches (CH) and those with no such history (NH). Patients with physician-diagnosed and managed post-traumatic headaches and migraines were all included in the “chronic headaches” cohort. Though the mechanisms underlying the development of these two diseases are different, definitive clinical diagnosis one way or another can be difficult [26, 27], especially among pediatric athletes involved in high contact sports. This difficulty can be attributed to the significant phenotypic overlap between the two conditions, including throbbing headache, pain on the side of the head, nausea, vomiting, and headache exacerbated by activity [28]. Additionally, pediatric patients report their symptoms and history of present illness less reliably, further increasing the difficulty of definitive diagnosis. Patients with chronic headaches of unspecified classification were also included in the analysis. In the clinical setting these cases should be followed longitudinally to determine and confirm an exact diagnosis. Given our study design, we lacked this privilege. However, excluding these cases would provide only a partial account of the chronic headache burden among the pediatric athlete sub-population [4]. Appropriate sample sizes for each cohort were derived using the equation below.

$$n=\frac{{\lbrack z_\alpha\sqrt{2PQ}+z_\beta\sqrt{p_1q_1+p_2q_2}\rbrack}^2}{{(p_1-p_2)}^2},where\;P=\frac{x_1+x_2}{n_1+n_2}and\;Q=1-P$$

According to the power analysis, we needed at least 524 patients in each group to detect a mean concussion incidence difference of 10% between the CH and NH cohorts, at 80% power and with a Type I error set at 0.05.

Chi-squared tests were used to compare categorical variables and concussion incidence. Continuous variables were presented as the mean ± standard deviation if they were normally distributed according to the Kolmogorov-Smirnov test. Means of normally distributed continuous variables were compared using a *t*-test. If not normally distributed, continuous variables were presented as the median and interquartile range. Medians were compared using the Kruskal-Wallis test. Significant univariate variables were included in a multivariate logistical regression to assess for risk factors of chronic headaches. Pertinent risk factors, specifically a history of concussions, were further analyzed as a function of chronic headache and symptom burden, as reported on the PCSS survey administered at the beginning of the season. Headache burden was rated on an overall scale ranging from 0 to 54 that assessed the intensity of headache, vomiting, nausea, balance, dizziness, sensitivity to light, sensitivity to noise, numbness, and visual changes on independent scales of 0 to 6. Symptom burden included all 22 measures on the baseline PCSS survey. For the analysis, past concussions were stratified by their number and characteristics, including confusion, anterograde amnesia, retrograde amnesia, and loss of consciousness.

Next, a multivariate model accounting for variables shown to modulate chronic headaches was used to compare the incidence of future concussion among those reporting a history chronic headache and those reporting no such history. Future concussion incidence was also analyzed as a function of initial headache burden. Separate multivariable logistic regressions were then conducted to assess for the role of chronic headaches on concussion severity and recovery as measured by deviations from baseline to PI and then to FU in the five composite scores. In the multivariate model assessing concussion recovery loss to FU, latency to FU, and deviations from baseline to PI in the five composite scores were included as confounding variables. Latency to FU was defined as the time elapsed between the PI and FU tests. A final set of multivariable linear regressions were used to assess the role of baseline demographic variables and headache burden on concussion severity and recovery.

A *p*-value < 0.05 was considered significant for all tests. RStudio 3.6 (R Foundation for Statistical Computing) was used for data analysis and Adobe Illustrator 27.0 (Adobe Incorporated) was used for figure creation.

## Results

### Cohort demographics

A total of 7,453 student-athletes were followed for an average of 1.3 ± 0.6 person-years, with a sum total of 25,815 ImPACT assessments collected at baseline, post-injury, and follow-up. Patient demographics and incidence calculations were based on 1,260 baseline tests for those with chronic headaches (CH) and 10,303 baseline tests for those with no such history (NH) [Table 1]. Participants ranged in age from 12 to 22 years, with 90% of high school age between 14 and 18 years old. Age among the two cohorts was similar with a mean age of 15.4 years old (CH vs. NH, 15.3 ± 1.5 vs. 15.4 ± 1.6, respectively, *P =* 0.60). However, there were more females in the CH cohort (39.5% vs. 33.4%, *P* < 0.0001). Complete detail on sports played, concussion history, and neuropsychiatric comorbidities is provided in Table 1.

**Table 1:** Dichotomized Cohort Demographics

	Chronic Headaches *n* = 1260	No History of Headaches *n* = 10,303	*P* Value
Age	15.3 ± 1.5	15.4 ± 1.6	0.60
Grade Level	9.23 ± 1.60	9.08 ± 1.65	**0.002**
Gender			
Male	762 (60.5)	6860 (66.6)	**< 0.0001**
Female	498 (39.5)	3443 (33.4)	**< 0.0001**
Contact Sports	1019 (80.9)	7910 (76.8)	**0.001**
Football	504 (40.0)	4041 (39.2)	0.59
Higher Risk Position	363 (28.8)	2255 (21.9)	**< 0.0001**
Linesman	276 (21.9)	1572 (15.3)	**< 0.0001**
Wide Receiver	68 (5.40)	543 (5.27)	0.85
Cornerback	19 (1.51)	140 (1.35)	0.67
Lower Risk Position	134 (10.6)	951 (9.23)	0.11
Running Back/ Fullback	58 (4.60)	440 (4.27)	0.58
Quarterback	38 (3.02)	239 (2.32)	0.13
Tight End	24 (1.90)	143 (1.39)	0.15
Safety	14 (1.11)	129 (1.25)	0.67
Soccer	176 (14.0)	1268 (12.3)	0.09
Basketball	108 (8.57)	921 (8.94)	0.67
Lacrosse	78 (6.19)	628 (6.10)	0.89
Cheerleading/ Gymnastics	87 (6.90)	473 (4.59)	**0.0003**
Wrestling/ Boxing/ Martial Arts	48 (3.81)	375 (3.64)	0.76
Water Polo/ Diving	18 (1.43)	204 (1.98)	0.18
Limited Contact Sports	192 (15.2)	1620 (15.7)	0.20
Baseball/ Softball	76 (6.03)	735 (7.13)	0.15
Volleyball	94 (7.46)	664 (6.44)	0.17
Track and Field	22 (1.75)	221 (2.15)	0.34
Years of Experience	2.26 ± 2.54	2.11 ± 2.53	**0.04**
Games Missed	1.64 ± 3.49	0.64 ± 1.93	**< 0.0001**
History of Concussions			
0	673 (53.4)	7070 (68.6)	**< 0.0001**
1	344 (27.3)	1909 (18.5)	**< 0.0001**
2+	226 (17.9)	750 (7.28)	**< 0.0001**
Type of Historic Concussion			
Confusion	598 (47.5)	1826 (17.7)	**< 0.0001**
Anterograde Amnesia	375 (29.8)	1130 (11.0)	**< 0.0001**
Retrograde Amnesia	280 (22.2)	836 (8.11)	**< 0.0001**
Loss of Consciousness	219 (17.4)	650 (6.31)	**< 0.0001**
Neuropsychiatric Comorbidities			
Depression/ Anxiety	127 (10.1)	304 (2.95)	**< 0.0001**
ADHD	71 (5.63)	507 (4.92)	0.27
DLD	50 (3.97)	272 (2.64)	**0.007**
Dyslexia	37 (2.94)	189 (1.83)	**0.008**
Autism	11 (0.87)	33 (0.32)	**0.003**
Epilepsy	34 (2.70)	94 (0.91)	**< 0.0001**
Substance Use Disorder	12 (0.95)	13 (0.13)	**< 0.0001**
Special Education	38 (3.02)	174 (1.69)	**0.001**
Speech Therapy	114 (9.05)	613 (5.95)	**< 0.0001**
English as Primary Language	1251 (99.3)	10,243 (99.3)	0.57
Baseline ImPACT Composite			
Symptom Score	10.37 ± 13.59	5.04 ± 8.84	**< 0.0001**
Migraine Cluster	3.28 ± 0.23	1.31 ± 0.05	**< 0.0001**
Headache	1.08 ± 0.04	0.42 ± 0.009	**< 0.0001**
Vomiting	0.09 ± 0.01	0.04 ± 0.003	**< 0.0001**
Nausea	0.20 ± 0.02	0.09 ± 0.005	**< 0.0001**
Balance	0.24 ± 0.02	0.10 ± 0.005	**< 0.0001**
Dizziness	0.37 ± 0.03	0.14 ± 0.006	**< 0.0001**
Sensitivity to Light	0.43 ± 0.03	0.16 ± 0.006	**< 0.0001**
Sensitivity to Noise	0.31 ± 0.02	0.11 ± 0.005	**< 0.0001**
Numbness	0.18 ± 0.02	0.08 ± 0.004	**< 0.0001**
Visual	0.39 ± 0.03	0.17 ± 0.007	**< 0.0001**
Cognitive Cluster	2.78 ± 0.18	1.49 ± 0.04	**< 0.0001**
Fatigue	0.58 ± 0.03	0.34 ± 0.009	**< 0.0001**
Drowsiness	0.54 ± 0.03	0.29 ± 0.008	**< 0.0001**
Slowed Down	0.26 ± 0.03	0.15 ± 0.006	**< 0.0001**
Fogginess	0.22 ± 0.02	0.11 ± 0.005	**< 0.0001**
Concentration	0.71 ± 0.04	0.38 ± 0.009	**< 0.0001**
Memory	0.46 ± 0.03	0.22 ± 0.007	**< 0.0001**
Sleep Cluster	2.05 ± 0.11	1.18 ± 0.03	**< 0.0001**
Trouble Falling Asleep	0.77 ± 0.04	0.44 ± 0.01	**< 0.0001**
Sleeping More	0.40 ± 0.03	0.19 ± 0.007	**< 0.0001**
Sleeping Less	0.89 ± 0.04	0.55 ± 0.01	**< 0.0001**
Neuropsychiatric Cluster	2.03 ± 0.13	1.10 ± 0.03	**< 0.0001**
Irritability	0.51 ± 0.03	0.27 ± 0.008	**< 0.0001**
Nervousness	0.61 ± 0.04	0.35 ± 0.009	**< 0.0001**
Sadness	0.41 ± 0.03	0.21 ± 0.007	**< 0.0001**
More Emotional	0.50 ± 0.03	0.27 ± 0.009	**< 0.0001**
Verbal Memory	81.09 ± 0.68	81.66 ± 0.27	0.12
Visual Memory	69.38 ± 0.87	70.73 ± 0.35	**0.004**
Processing Speed	34.6 ± 0.45	33.9 ± 0.18	**0.004**
Reaction Time	0.64 ± 0.06	0.64 ± 0.02	0.90

*ADHD *Attention deficit/ hyperactivity disorder, *DLD* Diagnosed learning disability, *ImPACT* Immediate post-concussion assessment and cognitive testing

Baseline Verbal Memory and Reaction Time ImPACT composite scores were similar between the two cohorts [Table 1]. However, the CH cohort had greater Symptom scores at baseline (10.37 ± 13.59 vs. 5.04 ± 13.58, *P <* 0.0001), worse Visual Memory scores (69.4 ± 0.87 vs. 70.7 ± 0.35, *P =* 0.004), and slower Processing Speeds (34.6 ± 0.45 vs. 33.9 ± 0.18, *P =* 0.004). Differences in baseline Symptom burden were prominent in all four Symptom clusters, including the Migraine, Cognitive, Sleep, and Neuropsychiatric clusters, as well as all 22 symptom measures.

#### Risk factors of chronic headaches

**Fig. 2:**
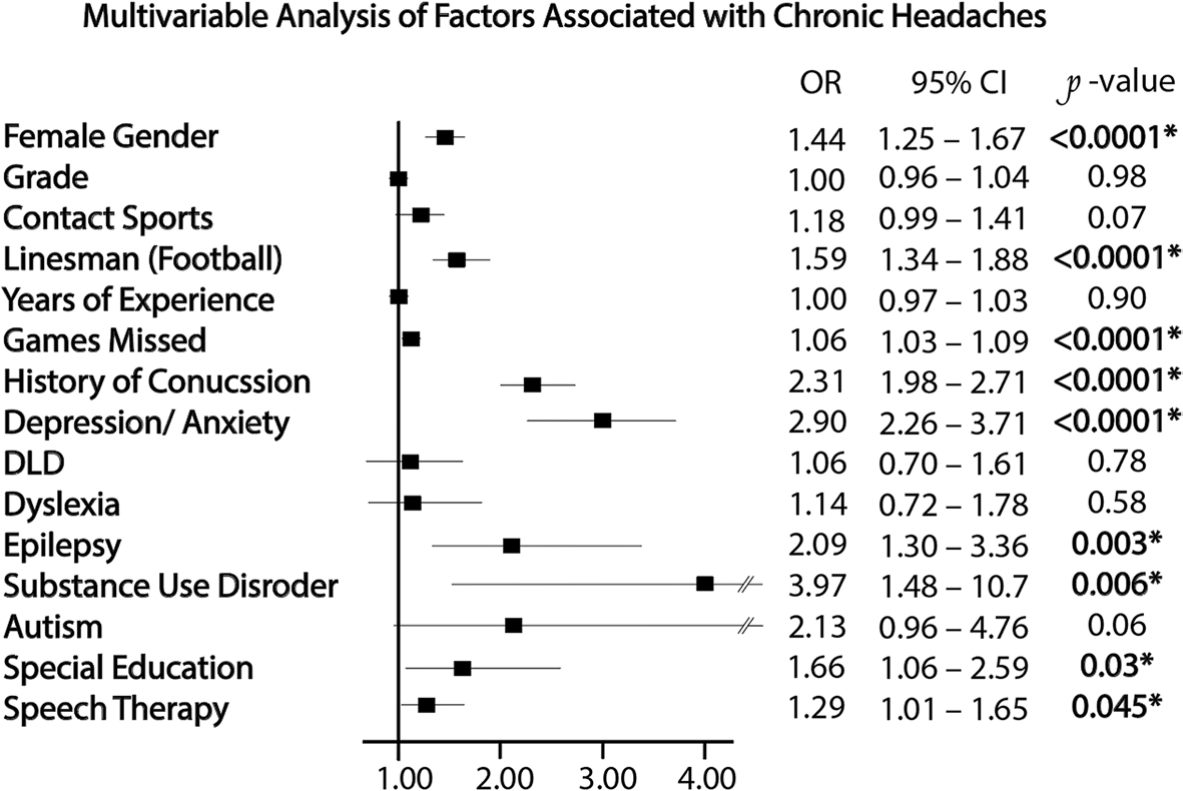
Multivariable Analysis of Factors Associated with Chronic Headaches

One thousand one hundred forty-seven patients reported chronic headaches (15.4%). On multivariate analysis female gender, lineman position, games missed during the season, a history of concussion, depression/ anxiety, epilepsy, substance use disorder, special education, and speech therapy were all independently associated with chronic headaches [Fig. 2]. Of these variables, female gender (OR 1.44, 95%CI [1.25–1.67]), lineman position (OR 1.59 [1.34–1.88]), a history of concussion (OR 2.31 [1.98–2.71]), and depression/ anxiety (OR 2.90 [2.26–3.71]) had both a notable magnitude of effect and considerable statistical significance (*P* < 0.0001).

Next, the relationship between a history of concussions and chronic headaches was further explored. In brief, for those with a history of multiple concussions, each additional concussion was correlated with progressively greater headache [Supplementary Fig. 2 A, *r*^2^ = 0.95] and symptom burden [Supplementary Fig. 2B, *r*^2^ = 0.94]. Moreover, a history of more severe concussions, including loss of consciousness concussions, was also associated with greater headache [Supplementary Fig. 2 C] and symptom burden [Supplementary Fig. 2D].

#### Concussion incidence and severity

**Fig. 3:**
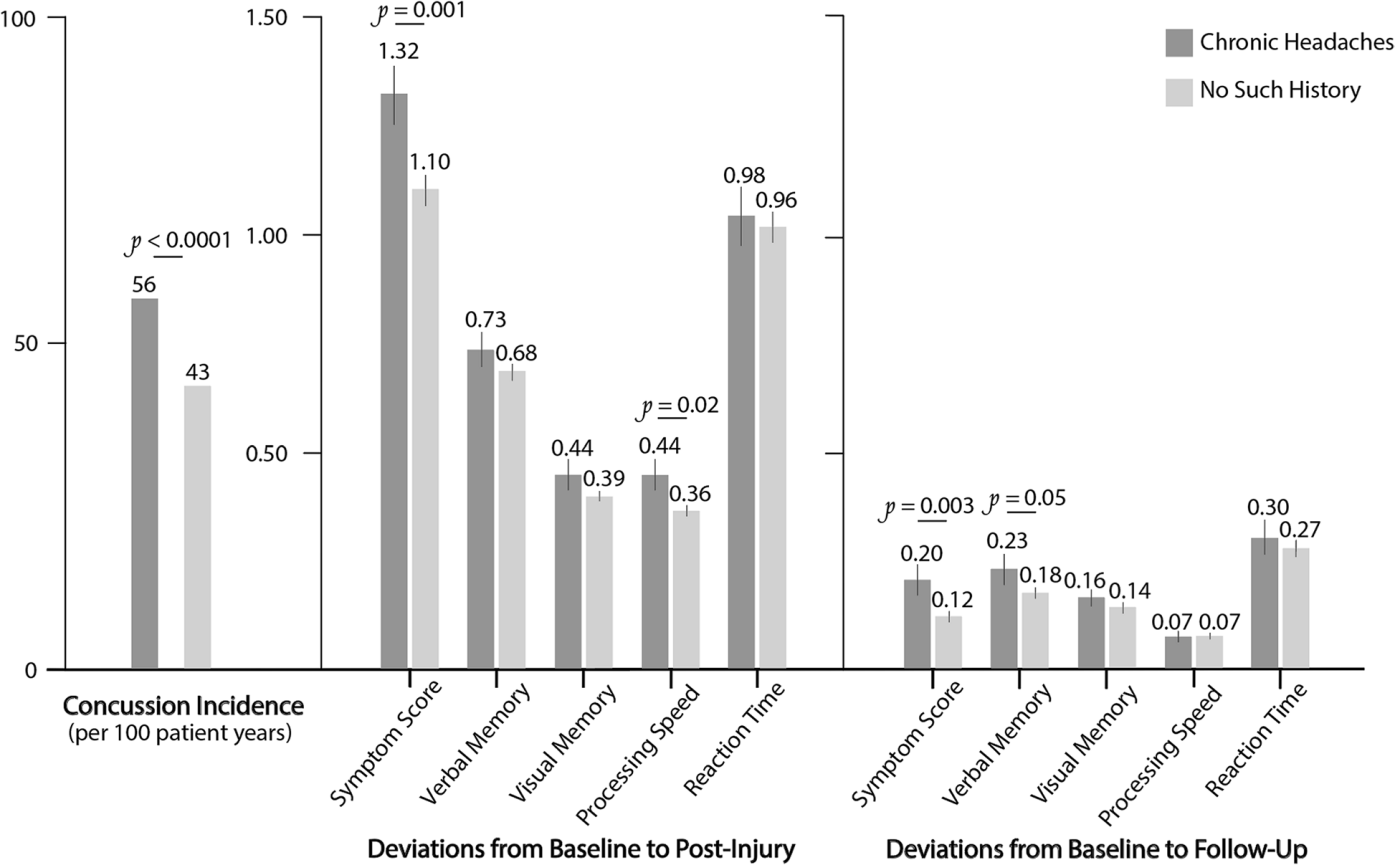
Concussion Incidence, Severity, and Recovery Dichotomized by Chronic Headache Status. Severity and recovery are presented as deviations in individual ImPACT composite scores from baseline to post-injury and follow-up, respectively

Incidence of future concussion among those with chronic headaches was 55.6 concussion per 100 patient years [Fig. 3]. In contrast, future concussion incidence was lower among those that did not have chronic headaches, at 43.0 concussions per 100 patient years (*P <* 0.0001). Next, we used deviations from baseline to PI symptoms and neurocognitive measures as a proxy to assess for concussion severity. Deviations from baseline to PI in Verbal, Visual Memory and Reaction Time were not different between the two cohorts. However, deviations from baseline to PI in overall Symptom burden were greater in the CH cohort as compared to the NH cohort (1.32 ± 0.07 vs. 1.10 ± 0.03, *P =* 0.001), as were deviations in Processing Speed (0.44 ± 0.03 vs. 0.36 ± 0.01, *P =* 0.02). Specifically, there were significant differences in deviations from baseline to PI in four separate Symptom measures, including headaches, irritability, sleeping more, and sleeping less [Supplementary Table 1].

**Fig. 4:**
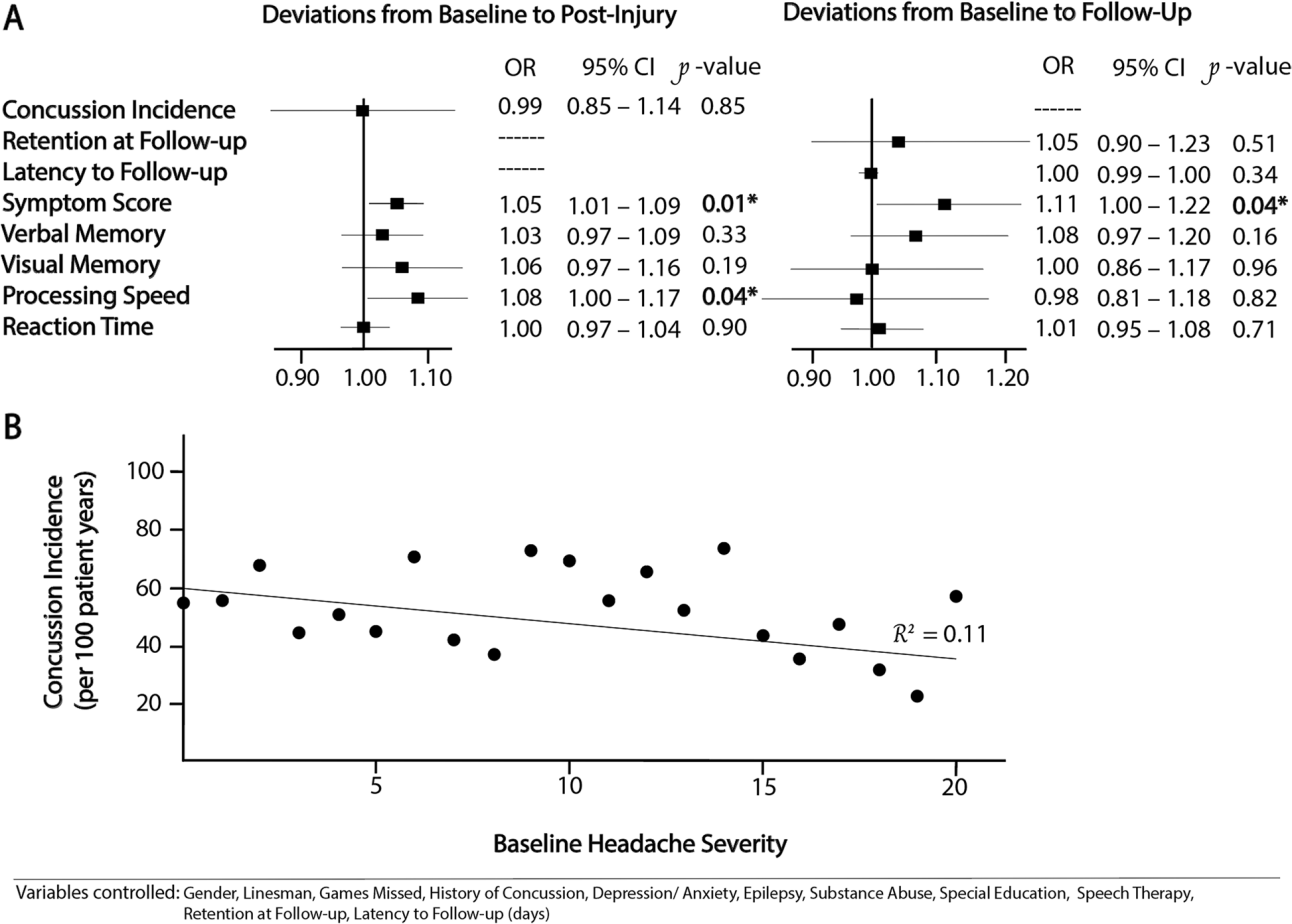
Multivariable Analysis of Concussion Incidence, Severity, and Recovery’s Association with Chronic Premorbid Headaches (**A**). Concussion Incidence as Function of Baseline Headache Severity (**B**)

Multivariate analysis controlling for variables shown to be associated with a history of chronic headaches, including gender, depression/ anxiety, and a history of concussion, demonstrated a premorbid history of headaches was no longer associated with an increased incidence of future concussions (OR 0.99 [0.85–1.14], *P* = 0.85) [Fig. 4 A]. Moreover, baseline symptom burden was not directly correlated to future concussion incidence in a stepwise manner [Fig. 4B, *r*^2^ = 0.11]. As initial symptom burden increased, concussion incidence remained stable from 56 concussions per 100 patient years at a baseline headache severity of 1 to 56 concussions per 100 patient years at a baseline headache severity greater than 20.

Next, multivariable models confirmed that there were greater deviations from baseline to PI Symptom scores (OR 1.05 [1.01–1.09], *P =* 0.01) and Processing Speeds (OR 1.08 [1.00-1.17], *P* = 0.04, respectively) in the CH cohort as compared to the NH cohort [Fig. 4 A]. Though other neurocognitive measures trended in the same direction, differences between the two cohorts in Verbal Memory, Visual Memory, and Reaction Time were not statistically significant.

#### Concussion recovery

Among the two cohorts, there was no difference in the percentage of students who returned for follow-up (CH vs. NH, 71% vs. 70%, respectively *P =* 0.99), nor in the time elapsed between PI and FU testing (7 days, IQR [5–13] vs. 7 days [5–13], *P =* 0.90). When controlling for demographic variables and PI symptom and neurocognitive composite scores, we still found no significant difference between the two cohorts in retention at follow-up or latency to follow-up [Fig. 4 A]. As expected, by FU both cohorts recovered significantly from PI neurocognitive and symptom sequelae [Fig. 3, *P* < 0.0001]. Next, we used deviations from baseline to FU symptoms and neurocognitive measures as a proxy to assess for recovery. When comparing the two cohorts at FU, the CH cohort retained elevated deviations from baseline in Symptom score as compared to the NH cohort, even when deviations from baseline to PI Symptom scores, our proxy for concussion severity, was accounted for in the multivariate model (OR 1.11 [1.00-1.22], *p* = 0.04) [Fig. 4 A]. Whereas none of the 22 symptom measures had normalized to baseline levels in the CH cohort, vomiting (-0.02 ± 0.006), insomnia (-0.09 ± 0.05), nervousness (-0.04 ± 0.02), and sleeping less than usual (-0.17 ± 0.02) were statistically lower than at baseline in the NH cohort.

Multivariate analysis showed female gender was independently associated with increased deviations from baseline to PI and FU Symptom scores in the CH cohort (OR 1.63, [1.23–2.15], *P* = 0.0007, OR 1.13, [1.01–1.27], *P* = 0.04, respectively) [Supplementary Table 2]. A similar trend was seen in FU Symptoms scores of those with chronic headaches characterized by dizziness (OR 1.08 [1.00-1.17], *P* = 0.048). However, other baseline headache characteristics including vomiting, nausea, imbalance, and sensitivity to noise or light were not more or less likely to be associated with persistently high Symptoms scores at PI and FU. Deviations from baseline to FU in all neurocognitive measures on ImPACT, including Verbal Memory, Visual Memory, Processing Speed, and Reaction Time, were similar between the two cohorts [Fig. 4 A].

## Discussion

This study sought to assess the relationship between chronic headaches and concussions, analyzing the role of historic concussions on chronic headaches, as well as that of premorbid headaches on future concussion incidence, severity, and recovery. We found a history of concussions was a significant contributor to headache burden among adolescents and young adults. However, those with chronic headaches were not more likely to be diagnosed with a future concussion, despite presenting with more severe concussions that had protracted recovery. Our findings not only suggest the need for conservative management among adolescents and young adults with chronic headaches, it also indicates a potential health care gap in this population. Those with chronic headaches may be referred for concussion diagnosis and management at lower rates than those with no such comorbidities.

The prevalence of chronic headaches among our cohort of student-athletes was 15%. High-quality studies estimate a 5 to 6% prevalence of chronic headaches among the general population [1, 2]. A longitudinal national survey out of Norway found 6% of young adults aged 26 to 28 experienced headaches multiple times a week [1]. A narrative review found a 5% prevalence of chronic headaches in 59 of 347 studies analyzed [2]. Specifically among subjects from 14 studies aged 10 to 19 the prevalence of chronic headaches was 3%. We report a 15% prevalence of chronic headaches among a group of student-athletes with an average age of 15. Our cohort may have a higher incidence of chronic headaches because it only includes student-athletes. Even though our analysis of a specific sub-population may not represent the national chronic headache burden among all adolescents, our findings are relevant to an increasingly athletic youth population, with approximately 60% of American adolescents involved in school-affiliated youth sports [8].

### Historic concussions as a risk factor for chronic headaches

We found that chronic headaches were independently associated with female gender (OR 1.44, 95%CI [1.25–1.67], *P* < 0.0001), depression/ anxiety (OR 2.90 [2.26–3.71], *P* < 0.0001), positioning as a lineman (OR 1.59 [1.34–1.88], *P* < 0.0001), and a history of concussions (OR 2.31 [1.98–2.71], *P*< 0.0001). Female gender and depression/ anxiety have both been previously implicated in large-scale studies as significant contributors to the national pediatric headache burden [5, 6]. Though chronic headaches are well-reported as post-concussion sequalae [7], a history of concussion has not been similarly implicated in large-scale studies [5, 6]. We found a history of concussions had a greater magnitude (OR 2.31) of effect in its association with chronic headaches than gender (OR 1.44), but not greater than depression or anxiety (OR 2.90), likely making it a moderate contributor to the national pediatric headache burden. Specifically, among those with chronic headaches, a greater number of past concussions (*r*^2^ = 0.95) as well as concussions characterized by loss of consciousness (*P* < 0.0001) were associated with more severe chronic headache and symptom burden. Our findings strengthen the correlation between the two conditions, demonstrating that a history of more severe and repetitive head trauma likely significantly contribute to adolescent headache burden.

We included patients with both post-traumatic headaches and primary migraines in the “chronic headaches” cohort. We believed this would increase the external validity of our findings, especially in clinical settings, where the classification of headache, whether post-traumatic or primary migraine, is not always clear [26–28]. Additional sub-group analysis demonstrated that those with chronic headaches classified as primary migraines by physicians also had a significant history of concussion (OR 1.81) [Supplementary Table 3], though to a lesser extent than the entire chronic headaches group (OR 2.31). Even though this does not mean concussions were the cause or primary contributing factor to these patients’ migraines, it suggests head trauma may play a role in the development of physician-diagnosed primary migraines, or vice versa. This piece of evidence adds to the cannon of literature emphasizing the difficulty of differentially diagnosing primary migraines and post-traumatic headaches as defined by the ICHD-3 [26–28]. A more specific definition for post-traumatic headaches may be warranted.

### Concussion incidence and severity

Though we initially found the CH cohort had a greater incidence of future concussions than the NH cohort (56 vs. 43 concussions per 100 patient years, *P* < 0.0001), multivariate analysis controlling for significant demographic, clinical, and sport-related variables yielded no such effect (OR 0.99 [0.85–1.14], *P* = 0.85). Additionally, more severe chronic headaches were not associated with increased incidence of future concussion (*r*^2^ = 0.11), further strengthening the lack of correlation. However, the CH did have greater deviations in Symptom Score from baseline to PI (OR 1.05 [1.01–1.09], *P =* 0.01), suggesting greater concussion severity as compared to the NH cohort. Increased severity seems to be driven by more severe headaches (1.73 ± 0.06 vs. 1.58 ± 0.03, *P =* 0.05), more irritability (0.60 ± 0.06 vs. 0.47 ± 0.02, *P =* 0.03), sleeping more (0.48 ± 0.06 vs. 0.38 ± 0.02, *P* = 0.05), as well as sleeping less (0.22 ± 0.06 vs. -0.06 ± 0.02, *P <* 0.0001).

In summary, there was comparable incidence of future concussion among the two cohorts, but greater increase in deviations from baseline to post-injury in symptom burden among those with chronic headaches. Multiple plausible explanations could unify these seemingly disparate findings. First, clinicians may simply have a greater symptom threshold to make the clinical diagnosis of concussion among those with premorbid headaches. Post-concussion symptoms are difficult to distinguish from the day-to-day symptoms those with chronic headaches experience, creating a potential for missed diagnoses and mismanagement of head trauma. In fact, among our patient cohort all 22 classic post-concussive symptoms were elevated at baseline in athletes with chronic headaches as compared to their peers. The challenge in distinguishing post-concussive sequelae from symptoms of premorbid conditions, such as depression and anxiety, has been described before [29, 30]. So long as post-traumatic symptomatic evaluation remains the gold-standard for concussion diagnosis, those with a history of headaches or other neuropsychiatric comorbidities may be prone to clinical anchoring bias of their baseline morbidities as the most plausible explanation of their post-traumatic symptoms [31, 32], leading to underdiagnosis of concussion and therapeutic mismanagement. Second, coaches and athletic staff may sideline those with a history of chronic headaches during intense, high stakes play, resulting in fewer minutes per season for those athletes to be at risk for head trauma [33]. Indeed, chronic headaches were independently associated with more games missed throughout the season (OR per game missed 1.06 [1.03–1.09], *P* < 0.0001), and when the number of games missed was controlled in the multivariate analysis for future concussion incidence, chronic headaches were no longer associated with an increased risk for future concussion. However, as cautious coaches manage this population of students conservatively, one would expect lower symptomatic and neurocognitive thresholds to concussion diagnosis. Greater, rather than lower, deviations from baseline to post-injury symptomology in this study’s cohort of concussed athletes with chronic headaches makes this explanation less likely.

The most salient positive risk factors for future concussions included participation in contact sports, fewer years of experience in the sport, and a history of previous concussions [Supplementary Table 2]. Previous concussions could put athletes at a greater risk for future concussion because of incomplete recovery, the tendency to be careless on the field, and a lack of situational awareness. This finding could also explain why multivariate models found chronic headaches were not independently associated with future concussions, in that having a history of past concussions more specifically predicts for future concussions. However, supplemental analysis showed a history of past concussions was independently associated with fewer rather than greater deviations from baseline to post-injury Symptoms scores, Processing Speeds, and Reaction Times [Supplementary Table 4]. Less rather than more severe concussions among previously concussed athletes make explanations of past concussions modulating future concussion risk less likely in our cohort. Rather, there may once again be a differential threshold to concussion diagnosis. Specifically, providers may be more cautious in the medical management of those with a history of diagnosed concussions, having a lower threshold to concussion diagnosis in this patient population, secondary to confirmation and anchoring biases of their medical history [31, 32].

In the end, it seems that though concussions may be significant contributors to the national adolescent headache burden, premorbid headaches themselves may not be an independent risk factor for concussion, potentially because of greater clinician threshold to diagnosis, especially considering premorbid headaches were associated with more severe concussions. Though the effect of premorbid headaches on concussion trends has not been studied extensively, preliminary evidence among Canadian ice hockey players in early adolescence suggested that preseason reports of dizziness, headaches, and neck pain were associated with increased concussion incidence [34]. Importantly, patients were asked about these symptoms at one point at the beginning of the season. Subjects were not questioned on the chronicity or severity of these symptoms. The patients in the present study were specifically asked about headaches diagnosed and treated by a physician and were asked to quantify their chronic symptom burden. Furthermore, the Canadian study was limited to ice hockey and only controlled for six demographic and sports-related variables in the multivariable analysis. Our multivariable analysis accounted for 15 of more than 50 demographic, clinical, academic, and sports-related variables that demonstrated significance in univariate analysis and included athletes from a wide range of youth sports.

### Concussion recovery

Those with chronic headaches had greater deviations in Symptom Score from baseline to FU (OR 1.11 [1.00-1.22], *P* = 0.04), suggesting impaired symptomatic recovery as compared to those without chronic headaches. Specifically, females (OR 1.13 [1.01–1.27], *P* = 0.04) and those with headaches characterized by dizziness at baseline (OR 1.08 [1.00-1.17,] *P* = 0.048) were more likely to have severe concussions and impaired recovery. Moreover, those with no history of chronic headaches, reported certain symptoms at follow-up that were even better than at baseline, including vomiting (-0.02 ± 0.006), insomnia (-0.09 ± 0.05), nervousness (-0.04 ± 0.02), and sleeping less than usual (-0.17 ± 0.02). However, those with a history of chronic headaches had no such improvements.

Given significant symptom overlap between chronic headaches and post-concussive sequela, recovery among this patient cohort presents new challenges. School aged girls with premorbid headaches have shown to take significantly longer to recover from a concussion [11]. We confirm such trends in the adolescent and young adult population. Those with a history of premorbid headaches, especially females and those whose headaches were characterized by dizziness at baseline, were less likely to recover to baseline symptomatology at a median of 7 days post-concussion than those with no such history. The greater symptomatic severity of concussions at post-injury among those with a history of premorbid concussions may also play a role in protracted recovery, however this variable was controlled in the multivariable analysis. Multiple premorbid conditions, including depression/ anxiety [23] and a history of prior concussions [35], have also shown to impede recovery, however both comorbidities were also controlled in the adjusted analysis. It seems head trauma increases the severity of chronic headaches longitudinally, impeding recovery to baseline levels.

### Clinical implications

Our findings suggest sports-related concussions have likely become a significant contributor to the national adolescent headache burden. Now more than ever post-concussion syndrome ought to be on the differential when assessing chronic headaches in both male and female youth, especially given its specific management paradigms, ranging from counseling return to athletic participation, especially at high-risk on-field positions, to management of its neuropsychiatric manifestations, such as depression and anxiety [36]. It does not help that post-concussion syndrome has been reported as prevalent and vastly underdiagnosed in the pediatric population, and that it seldom resolves spontaneously, remaining persistent even 60 months after concussion [37]. Moreover, with promising therapies for post-concussion syndrome such as hyperbaric oxygen in the clinical trial pipeline [38], recognizing the role of concussions on the national adolescent headache burden will be vital for proper management of pediatric headaches into the future.

Next, though we found athletes with chronic headaches were more likely to be sidelined during the season, recognizing that head trauma confers more severe concussions with protracted recovery in this patient population should encourage even more conservative counseling on return to play, especially among females and those with headaches characterized with dizziness.

Finally, we hypothesized that concussions may be underdiagnosed among youth with chronic headaches, given significant symptom overlap between the two conditions. Specifically, we found clinicians referred patients with chronic headaches to post-injury testing when they had significantly greater deviations in symptoms from their baseline as compared to their peers. Clinical underdiagnosis of concussion can lead to mismanagement of head trauma, with continued engagement in scholarly and athletic responsibilities, loss of critical periods of rest, and earlier return to play [39]. Mismanagement of head trauma, in turn, can lead to significant long-term complications, including increased risk for further head trauma, second impact syndrome, and post-concussion syndrome [40]. Of note, unlike the Symptom composite, the four neurocognitive ImPACT composite scores were either impaired to a similar extent at post-injury or marginally elevated in those with premorbid headaches as compared to those with no such history, suggesting similar neurocognitive concussion severity and/ or a comparable neurocognitive threshold to concussion diagnosis. Simply put, physicians may not be taking changes in verbal memory, visual memory, reaction time, or processing speed into consideration when making a clinical concussion diagnosis. As previously suggested [40–43], clinical concussion diagnosis supplemented with tests tracking changes in neurocognitive ability may help distinguish post-concussive symptom sequelae from chronic headache symptomology, potentially addressing some extent of underdiagnosis in this patient population. Interestingly, Verbal Memory and Reaction Time were not elevated among those with chronic headaches at baseline, meaning post-injury neurocognitive testing may be beneficial for diagnosis even with a lack of baseline testing to normalize the outcomes. Not only would appropriate diagnosis lead to better short-term and long-term clinical outcomes, it may also have implications for the health-care industry at large as well as insurance providers.

Of note, the CDC currently lists computerized cognitive testing as a moderate, Level C recommendation for mild traumatic brain injury among children [16]. Additionally, only 20% of American and a mere 1% of Canadian family practitioners have been reported using neurocognitive testing [44]. Our findings may support the increased use of validated, age-appropriate computerized cognitive testing among student-athletes with premorbid neuropsychiatric comorbidities such as chronic headaches.

### Strengths

This study included a large sample of adolescent and youth athletes from multiple sporting backgrounds accounting for over a decade of data. The data acquired was extensive with over 50 demographic and clinical variables, as well as more than 25,000 ImPACT assessments. The data was acquired prospectively, and data collection was supervised by a national research-oriented organization. Of note, in those that endorsed or denied chronic headaches at baseline, only 14 patients had missing/ incomplete data in any of the 50 + variables at baseline, meaning 99.8% of baseline tests had complete data, suggesting rigorous quality assurance. To our knowledge, this is the first study to examine the role of historic concussions, as well as the incidence of future concussions and concussion severity among adolescents and young adults with premorbid chronic headaches.

### Limitations

There are multiple limitations worthy of acknowledgement. First, concussions were not always initially examined by physicians. Other members of the athletic staff, including physiotherapists and athletic trainers also evaluated head injury. Though the gold-standard remains physician diagnosis of concussion, initial examination of head trauma on the field is typically performed by a variety of athletic staff and midlevel providers who are trained in concussion management, bolstering the external validity of our study. If students are to continue to be examined by non-physicians, then it is imperative that all members of the adolescent health care team understand that those with a history of chronic headaches may require more conservative management and may potentially be underdiagnosed. Second, though the data was prospectively collected in a well-organized electronic format and supervised by a research-oriented organization, the analysis was completed retrospectively. This means the database was not specifically curated for the question at hand. We could not include variables such as the months or years experiencing chronic headaches in our analysis. Finally, though a decade worth of data is an apparent strength of the analysis, we must acknowledge the significant changes in concussion diagnosis and management guidelines in this timeframe, including increased outpatient follow-up and return to play time [16]. Even so, the recent endpoint of the study (2019) bolsters its external validity.

## Conclusion

In a multi-center cohort of 7,453 youth athletes, a history of concussions was a significant contributor to headache burden among adolescents and young adults. However, those with chronic headaches were not more likely to be diagnosed with a concussion, despite presenting with more severe concussions that had protracted recovery. Our findings not only suggest the need for conservative management among youth athletes with chronic headaches, it also indicates a potential health care gap in this population, in that those with chronic headaches may be referred for concussion diagnosis and management at lower rates than those with no such comorbidity.

## Supplementary Information

**Additional file 1:** **Supplementary Table 1. **Changes from Baseline to Post-Injury and Follow-up Symptoms Measures. **Supplementary Table 2. **Multivariable Analysis of Factors Associated with Chronic Migraines. **Supplementary Table 3. **Multivariable Analysis of Factors Associated with Future Concussion Incidence, Severity, and Recovery. **Supplementary Table 4. **Multivariable Analysis of Concussion Severity among Student-Athletes with Past Concussions.
