# Supplementary material for: A multicenter, longitudinal survey of headaches and concussions among youth athletes in the United States from 2009 to 2019
Source: J Headache Pain. 2023 Feb 8;24(1):6. doi: 10.1186/s10194-022-01528-3 (PMC9909942; doi:10.1186/s10194-022-01528-3)
Supplement: Supplementary file 1 — Additional file 1: Supplementary Table 1. Changes from Baseline to Post-Injury and Follow-up Symptoms Measures. Supplementary Table 2. Multivariable Analysis of Factors Associated with Chronic Migraines. Supplementary Table 3. Multivariable Analysis of Factors Associated with Future Concussion Incidence, Severity, and Recovery. Supplementary Table 4. Multivariable Analysis of Concussion Severity among Student-Athletes with Past Concussions. [file 10194_2022_1528_MOESM1_ESM.docx]

**SUPPLEMENTARY MATERIAL**

**A Multicenter, Longitudinal Survey of Headaches and Concussions Among Youth Athletes in the United States from 2009 to 2019**

Muhammad Ali^1^, BA, Nek Asghar^1^, DO, Theodore Hannah^1^, MD, Alexander J Schupper^1^ MD, Adam Li^1^, MD, Nickolas Dreher^1^, MD, Muhammad Murtaza-Ali^1^, Vikram Vasan^1^, BS, Zaid Nakadar^2^, BS, Husni Alasadi^1^, BS, Anthony Lin^3^, BS, Eugene Hrabarchuk^1^, BS, Addison Quinones^1^, BS, Lily McCarthy^1^, BS, Zerubabbel Asfaw^1^, BS, Jonathan Dullea^1^, MPH, Alex Gometz^2^, DPT, Mark Lovell^3^, PhD, and Tanvir Choudhri^1^, MD

**Affiliations:**

^1^ Department of Neurosurgery, Icahn School of Medicine at Mount Sinai, NY, NY, USA, 10021

^2^ Department of Medical Education, State University of New York Downstate Health Sciences University, NY, NY, USA, 11203

^3^ Department of Medical Education, Joan & Sanford I. Weill Medical College of Cornell University, NY, NY, USA, 10021

^4^ Concussion Management of New York, NY, NY, USA, 10021

^5^ Department of Neurology, The University of Pittsburgh Medical Center, Pittsburgh, PA, USA, 15260

**Address correspondence to:**

Muhammad Ali, Department of Neurosurgery, Icahn School of Medicine at Mount Sinai

muhammad.ali@icahn.mssm.edu

518-577-1783

**Supplementary Tables:** 4

**Supplementary Figures:** 2

**Supplementary Table 1|** Changes from Baseline to Post-Injury and Follow-up Symptoms Measures

|  | Post-Injury | | | | Follow-Up | | | | |  |
| --- | --- | --- | --- | --- | --- | --- | --- | --- | --- | --- |
|  | CH | NH | | *P* Value | CH | | NH | | *P* Value | |
| Migraine Cluster | 6.38 ± 0.32 | 6.14 ± 0.11 | | 0.48 | 1.14 ​​± 0.47 | | 1.59 ± 0.10 | | 0.28 | |
| Headache | 1.73 ± 0.06 | 1.58 ± 0.03 | | **0.05** | 0.38 ± 0.12 | | 0.39 ± 0.03 | | 0.91 | |
| Vomiting | 0.03 ± 0.02 | 0.05 ± 0.007 | | 0.44 | -0.04 ± 0.04 | | **-0.02 ± 0.006** | | 0.31 | |
| Nausea | 0.50 ± 0.05 | 0.51 ± 0.02 | | 0.94 | -0.01 ± 0.05 | | 0.08 ± 0.01 | | 0.07 | |
| Balance | 0.65 ± 0.05 | 0.66 ± 0.02 | | 0.77 | 0.12 ± 0.07 | | 0.20 ± 0.02 | | 0.18 | |
| Dizziness | 0.92 ± 0.06 | 0.89 ± 0.02 | | 0.60 | 0.08 ± 0.07 | | 0.22 ± 0.02 | | **0.04** | |
| Sensitivity to Light | 1.04 ± 0.06 | 1.04 ± 0.02 | | 0.95 | 0.29 ± 0.09 | | 0.32 ± 0.02 | | 0.77 | |
| Sensitivity to Noise | 0.91 ± 0.06 | 0.89 ± 0.02 | | 0.69 | 0.25 ± 0.08 | | 0.29 ± 0.02 | | 0.60 | |
| Numbness | 0.17 ± 0.03 | 0.15 ± 0.01 | | 0.45 | -0.03 ± 0.05 | | -0.01 ± 0.01 | | 0.70 | |
| Visual | 0.41 ± 0.05 | 0.37 ± 0.02 | | 0.38 | 0.10 ± 0.08 | | 0.11 ± 0.02 | | 0.84 | |
| Cognitive Cluster | 5.08 ± 0.28 | 4.78 ± 0.10 | | 0.31 | 1.29 ± 0.39 | | 1.44 ± 0.10 | | 0.72 | |
| Fatigue | 0.73 ± 0.07 | 0.66 ± 0.02 | | 0.34 | 0.06 ± 0.09 | | 0.13 ± 0.02 | | 0.46 | |
| Drowsiness | 0.82 ± 0.06 | 0.78 ± 0.02 | | 0.57 | 0.19 ± 0.08 | | 0.19 ± 0.02 | | 0.97 | |
| Slowed Down | 0.77 ± 0.05 | 0.73 ± 0.02 | | 0.42 | 0.24 ± 0.07 | | 0.21 ± 0.02 | | 0.81 | |
| Fogginess | 0.83 ± 0.06 | 0.85 ± 0.02 | | 0.79 | 0.25 ± 0.07 | | 0.29 ± 0.02 | | 0.61 | |
| Concentration | 1.00 ± 0.07 | 0.97 ± 0.02 | | 0.59 | 0.38 ± 0.10 | | 0.35 ± 0.02 | | 0.73 | |
| Memory | 0.64 ± 0.05 | 0.54 ± 0.02 | | 0.08 | 0.17 ± 0.09 | | 0.26 ± 0.02 | | 0.26 | |
| Sleep Cluster | 1.72 ± 0.12 | 1.22 ± 0.04 | | **<0.0001** | 0.06 ± 0.22 | | **-0.09 ± 0.05** | | 0.45 | |
| Falling Asleep | 1.01 ± 0.06 | 0.81 ± 0.18 | | 0.08 | 0.13 ± 0.11 | | 0.02 ± 0.02 | | 0.25 | |
| Sleeping More | 0.48 ± 0.06 | 0.38 ± 0.02 | | **0.05** | -0.06 ± 0.07 | | 0.06 ± 0.02 | | 0.09 | |
| Sleeping Less | 0.22 ± 0.06 | -0.06 ± 0.02 | | **<0.0001** | -0.01 ± 0.10 | | **-0.17 ± 0.02** | | 0.10 | |
| Neuropsychiatric Cluster | 1.19 ± 0.16 | 0.93 ± 0.06 | | 0.11 | 0.29 ± 0.23 | | 0.12 ± 0.06 | | 0.47 | |
| Irritability | 0.60 ± 0.06 | 0.47 ± 0.02 | | **0.03** | 0.18 ± 0.08 | | 0.16 ± 0.02 | | 0.78 | |
| Nervousness | 0.15 ± 0.04 | 0.11 ± 0.02 | | 0.36 | -0.08 ± 0.08 | | **-0.04 ± 0.02** | | 0.65 | |
| Sadness | 0.22 ± 0.04 | 0.15 ± 0.02 | | 0.14 | 0.06 ± 0.07 | | -0.01 ± 0.02 | | 0.27 | |
| More Emotional | 0.23 ± 0.05 | 0.21 ± 0.02 | | 0.74 | 0.12 ± 0.07 | | 0.02 ± 0.02 | | 0.13 | |
| Irritability | 6.38 ± 0.32 | 6.14 ± 0.11 | | 0.48 | 1.14 ​​± 0.47 | | 1.59 ± 0.10 | | 0.28 | |
|  |  |  |  | |  |  | |  |  |  |

**Supplementary Table 2|** Multivariable Analysis of Factors Associated with Chronic Migraines

|  | OR | 95% CI | *P* Value |
| --- | --- | --- | --- |
| Female Gender | 1.30 | 1.01 – 1.67 | **0.04** |
| Grade | 1.06 | 1.02 – 1.12 | 0.06 |
| Contact Sports | 1.17 | 0.92 – 1.50 | 0.19 |
| Linesman | 1.00 | 0.81 – 1.23 | 0.99 |
| Years of Experience | 1.01 | 0.98 – 1.05 | 0.54 |
| Games Missed | 1.06 | 1.03 – 1.09 | **<0.0001** |
| History of Concussion | 1.80 | 1.46 – 2.22 | **<0.0001** |
| Depression/ Anxiety | 2.60 | 1.94 – 3.49 | **<0.0001** |
| DLD | 1.42 | 0.91 – 2.22 | 0.12 |
| Dyslexia | 1.08 | 0.68 – 1.74 | 0.74 |
| Epilepsy | 2.08 | 1.23 – 3.52 | **0.006** |
| Substance Use Disorder | 3.33 | 1.12 – 9.87 | **0.03** |
| Autism | 1.35 | 0.41 – 4.46 | 0.62 |
| Special Education | 1.16 | 0.66 – 2.03 | 0.60 |
| Speech Therapy | 1.40 | 1.03 – 1.90 | **0.03** |
| Abbreviation: DLD, Diagnosed Learning Disability | | | |

**Supplementary Table 3|** Multivariable Analysis of Factors Associated with Future Concussion Incidence, Severity, and Recovery

|  | OR | 95% CI | *P* Value | |
| --- | --- | --- | --- | --- |
| Factors Associated with Future Concussion Incidence | | | | |
| Female Gender | 1.17 | 1.06 – 1.30 | **0.002** | |
| Grade | 0.99 | 0.96 – 1.02 | 0.47 | |
| Contact Sports | 1.63 | 1.45 – 1.83 | **<0.0001** | |
| Linesman | 1.20 | 1.06 – 1.36 | **0.005** | |
| Years of Experience | 0.95 | 0.94 – 0.97 | **<0.0001** | |
| Games Missed | 1.01 | 0.99 – 1.04 | 0.29 | |
| History of Concussion | 2.31 | 2.03 – 2.62 | **<0.0001** | |
| Chronic Headaches | 0.99 | 0.85 – 1.14 | 0.85 | |
| Depression/ Anxiety | 0.98 | 0.77 – 1.23 | 0.84 | |
| DLD | 0.94 | 0.69 – 1.29 | 0.78 | |
| Factors Associated with Deviations in Symptom Score from Baseline to **Post-Injury** | | | | |
| Female Gender | 1.63 | 1.23 – 2.15 | **0.0007** | |
| Baseline Headache Burden | 0.93 | 0.83 – 1.04 | 0.17 | |
| Baseline Vomiting Burden | 0.86 | 0.65 – 1.14 | 0.29 | |
| Baseline Nausea Burden | 1.13 | 0.92 – 1.40 | 0.23 | |
| Baseline Imbalance Burden | 0.95 | 0.78 – 1.16 | 0.62 | |
| Baseline Dizziness Burden | 1.13 | 0.94 – 1.37 | 0.20 | |
| Baseline Sensitivity to Light | 0.96 | 0.83 – 1.11 | 0.57 | |
| Baseline Sensitivity to Noise | 0.93 | 0.78 – 1.10 | 0.39 | |
| Baseline Numbness Burden | 0.86 | 0.72 – 1.04 | 0.12 | |
| Baseline Visual Problems | 0.93 | 0.82 – 1.06 | 0.29 | |
| Factors Associated with Deviations in Symptom Score from Baseline to **Follow-up** | | | | |
| Female Gender | 1.13 | 1.01 – 1.27 | **0.04** | |
| Baseline Headache Burden | 1.00 | 0.96 – 1.05 | 0.17 | |
| Baseline Vomiting Burden | 0.97 | 0.86 – 1.17 | 0.29 | |
| Baseline Nausea Burden | 1.08 | 0.99 – 1.17 | 0.09 | |
| Baseline Imbalance Burden | 1.04 | 0.96 – 1.13 | 0.38 | |
| Baseline Dizziness Burden | 1.08 | 1.00 – 1.17 | **0.048** | |
| Baseline Sensitivity to Light | 0.98 | 0.92 – 1.04 | 0.43 | |
| Baseline Sensitivity to Noise | 0.99 | 0.92 – 1.06 | 0.80 | |
| Abbreviation: DLD, Diagnosed Learning Disability | | | |  |
|  |  |  |  | |

**Supplementary Table 4|** Multivariable Analysis of Concussion Severity among Student-Athletes with Past Concussions

|  | OR | 95% CI | *P* Value |
| --- | --- | --- | --- |
| Deviations from Baseline to **Post-Injury** |  |  |  |
| Symptom Score | 0.95 | 0.90 - 1.00 | **0.04** |
| Verbal Memory | 0.94 | 0.88 - 1.02 | 0.12 |
| Visual Memory | 0.91 | 0.81 - 1.02 | 0.10 |
| Processing Speed | 0.87 | 0.73 - 0.97 | **0.01** |
| Reaction Time | 0.94 | 0.90 - 0.99 | **0.02** |
| Variables controlled: Gender, Linesman, Games Missed, History of Concussion, Depression/ Anxiety, Epilepsy, Substance Abuse, Special Education, Speech Therapy, Retention at Follow-up, Latency to Follow-up (days) | | | |

**Supplementary Figure Legends**

**Supplementary Figure 1 | Flowchart of all Student Athletes Enrolled to the ImPACT Study Protocol**. Of 9,226 total student- athletes, 7,453 met all six inclusion ad exclusion criteria. Of these patients 5,214 presented for testing after concussion and were followed longitudinally.

**Supplementary Figure 2 | Chronic Symptom (A) and Headache (B) Burden as a Function of the Number of Past Concussions. Chronic Symptom (C) and Headache (D) Burden for each type of Historic Concussion: Confusion, Anterograde Amnesia, Retrograde Amnesia, and Loss of Consciousness (LOC).**
